# 

**Published:** 1886-07

**Authors:** 


					﻿slumber, 318.
JULY, I886.
Vol. LIII., Nurpbetd
THE
BO MEDICAL JOURNAL ANO EXAMINER
EDITORS:
S. J. JONES, M.D., LL.D.
W JAGGARD, A.M., M.D
HAROLD N. MOYER, M.D
PUBLISHED MONTHLY BY
THE OLARK & LONGLEY GO.,
Under direction of
THE CHICAGO MEDICAL PRESS ASSOCIATION,
308-316 Dearborn Street.
				

